# Nutritional behaviors and adaptation before and after metabolic bariatric surgery: a qualitative study based on patient experiences

**DOI:** 10.3389/fnut.2026.1866634

**Published:** 2026-09-09

**Authors:** Durdane Yılmaz Güven, Erkan Aksoy

**Affiliations:** 1 Department of Nursing, Faculty of Health Sciences, Karabuk University, Karabuk, Türkiye; 2 Department of Medical Services and Techniques, Operating Room Services Program, American University of Cyprus, Nicosia, Cyprus

**Keywords:** feeding behaviors, metabolic bariatric surgery, nursing care, patient experience, qualitative research

## Abstract

**Background:**

Although metabolic bariatric surgery is an effective treatment for obesity, qualitative evidence exploring patients’ experiences across both pre- and post-operative periods remains limited. This study aimed to examine patients’ decision-making experiences, nutritional behaviors, and perceptions of nursing care throughout this process.

**Methods:**

This qualitative descriptive study included individuals who had undergone metabolic bariatric surgery at least 12 months previously at a private hospital in Türkiye. Data were collected through focus group discussions (FGDs) followed by individual semi-structured interviews (ISIs) to obtain both shared and in-depth individual perspectives. All FGDs and ISIs were audio-recorded and transcribed verbatim. The FGD and ISI data were initially analyzed separately using inductive qualitative content analysis and subsequently compared and integrated during the later stages of analysis. Codes were grouped into categories, subthemes, and overarching themes through an iterative coding process. The coding process was independently reviewed by a second qualitative researcher, and discrepancies were resolved through consensus.

**Results:**

Five main themes emerged: decision-making for surgery, preoperative dietary habits, post-operative nutritional strategies, postoperative challenges, and information sharing. Participants reported that physical, psychological, and social factors influenced their decision to undergo surgery. Preoperatively, high-calorie and irregular eating patterns were common, whereas post-operative adaptation involved portion control, slow eating, and meal planning. Early postoperative challenges included physical symptoms, difficulties with dietary adherence, and limited social support. Structured education and regular nursing counseling were found to enhance patients’ confidence and facilitate adaptation.

**Conclusion:**

Metabolic bariatric surgery represents a comprehensive care process requiring physical, behavioral, psychological, and social adaptation throughout the surgical journey. The findings highlight the importance of structured nursing support, including individualized education, psychosocial guidance, and long-term follow-up, to facilitate patients’ adaptation after surgery.

## 1 Introduction

Obesity remains a major public health problem with a rapidly increasing prevalence worldwide and is closely associated with cardiovascular disease, type 2 diabetes, obstructive sleep apnea, and certain types of cancer (1). Current international guidelines recommend metabolic bariatric surgery as an effective and safe treatment option for individuals who do not achieve adequate weight loss through lifestyle modifications and pharmacological treatments (1, 2). Common surgical procedures, such as sleeve gastrectomy and Roux-en-Y gastric bypass, reduce stomach volume and alter gastrointestinal hormonal responses, resulting in significant changes in appetite, satiety, and food preferences. However, these biological effects alone may not be sufficient to ensure sustained outcomes; behavioral modification, patient education, and regular clinical monitoring remain important for maintaining long-term weight loss and metabolic improvements (2, 3).

Recommended nutritional protocols following metabolic bariatric surgery include adequate protein and fluid intake, a low-energy diet, and lifelong vitamin and mineral supplementation. Nevertheless, micronutrient deficiencies and nutrition-related complications remain important concerns in clinical practice, highlighting the need for regular laboratory monitoring, early identification, and appropriate supplementation (4–7).

While quantitative studies on eating behaviors provide important data on adherence and complications, qualitative research offers valuable insights into patients’ experiences and the contextual factors influencing adherence. Qualitative evidence indicates that portion control, regulation of eating speed, emotional eating, anxiety related to eating in social situations, and concerns about weight regain are frequently reported during the post-bariatric surgery period (3, 8). Beyond these behavioral challenges, psychosocial factors, including anxiety, emotional distress, self-efficacy, body image perceptions, and coping abilities, have been identified as important determinants of postoperative adaptation and long-term outcomes (9, 10). Patients may also experience uncertainty regarding postoperative success, fear of weight regain, and difficulties adapting to new eating behaviors, particularly during the early stages of recovery (3). In contrast, social support from family members, peers, and healthcare professionals has been associated with better adherence, psychological wellbeing, and weight-loss outcomes (11). Collectively, these findings highlight the importance of addressing behavioral, emotional, and psychosocial factors and providing appropriate professional support to facilitate adaptation to postoperative nutritional requirements and sustainable lifestyle changes (10).

A multidisciplinary team approach is essential in metabolic bariatric surgery care, with nurses assuming important responsibilities from preoperative education to long-term follow-up. Clinical guidelines emphasize nutritional education, regular nutritional assessment, and patient counseling as key components of postoperative management (12–14). Contemporary bariatric surgery guidelines also highlight the importance of coordinated interdisciplinary care involving surgeons, nurses, dietitians, psychologists, and other healthcare professionals in supporting patients’ nutritional, behavioral, and psychosocial adaptation throughout the surgical process (14, 15). Within this multidisciplinary framework, nurses contribute to patient education, monitoring, care coordination, and access to appropriate support services, while ongoing follow-up enables nutritional status to be monitored, potential deficiencies to be identified, and challenges related to dietary adaptation to be addressed (15).

Nutritional behaviors following metabolic bariatric surgery should therefore be understood not only in terms of physiological changes but also within the broader behavioral, psychological, social, and cultural context of postoperative adaptation. Patients may continue to experience challenges related to eating behaviors, self-regulation, social interactions, and long-term adaptation even years after surgery, underscoring the ongoing need for professional support and follow-up (16). In Türkiye, where communal eating practices and carbohydrate-rich dietary patterns are common, adaptation to postoperative dietary recommendations may involve additional challenges. Although previous research has explored patients’ experiences after bariatric surgery, relatively few qualitative studies have examined how nursing care, nutritional education, and interdisciplinary support contribute to patients’ nutritional adaptation within such cultural contexts. Therefore, this study aimed to explore patients’ decision-making experiences, nutritional behaviors, and perceptions of nursing care throughout the metabolic bariatric surgery process, from the preoperative to the postoperative period.

## 2 Materials and methods

### 2.1 Study design

This study employed a qualitative descriptive design to explore the experiences, nutritional behaviors, and adaptation processes of individuals before and after metabolic bariatric surgery. This design was considered appropriate because it enables an in-depth exploration of participants’ experiences and perspectives while remaining close to their accounts. A complementary data-collection approach combining focus group discussions (FGDs) and individual semi-structured interviews (ISIs) was used to capture both shared and individual experiences. FGDs facilitated interaction and collective reflection among participants, whereas ISIs provided an opportunity for a more detailed exploration of personal experiences that might not have been fully expressed in the group setting (17–20).

### 2.2 Study setting and time

The study was conducted between April and May 2021 at a private hospital in Türkiye where metabolic bariatric surgery was performed.

### 2.3 Participants and recruitment

Participants were recruited using purposive sampling. Individuals who had undergone metabolic bariatric surgery at the study hospital and met the eligibility criteria were directly approached by the researcher and invited to participate. The inclusion criteria were being 18 years of age or older, being at least 12 months postoperatively, and being able to read and write in Turkish. Individuals who met the eligibility criteria and voluntarily agreed to participate were included in the study. A fixed sample size was not predetermined; recruitment and data collection continued until data saturation was achieved. The final sample consisted of 13 participants. The demographic and clinical characteristics of the participants are presented in Table 1.

**TABLE 1:** Demographic and clinical characteristics of the participants.

Participant no.	Age	Gender	Educational level	Marital status	Job	Height	Weight before surgery	BMI	Weight after surgery	Duration after surgery
P1	32	Female	High school	Single	Nurse	1.65 cm	130 kg	47.8	74.8 kg	15 month
P2	33	Female	High school	Married	Nurse	1.63 cm	110 kg	41.4	70 kg	19 month
P3	30	Female	Master’s degree	Married	Academic	1.64 cm	120 kg	44.6	78 kg	18 month
P4	34	Female	High school	Married	Housewife	1.60 cm	108 kg	42.2	58 kg	24 month
P5	41	Male	Bachelor degree	Married	Tradesman	1.79 cm	134 kg	41.8	85 kg	24 month
P6	30	Female	Bachelor degree	Married	Lawyer	1.61 cm	112 kg	43.2	74 kg	17 month
P7	48	Female	Bachelor degree	Married	Engineer	1.54 cm	142 kg	59.9	82 kg	15 month
P8	35	Female	High school	Married	Housewife	1.50 cm	108 kg	48.0	64 kg	15 month
P9	44	Female	High school	Married	Housewife	1.60 cm	120 kg	46.9	65 kg	24 month
P10	45	Female	High school	Married	Coordinator	1.62 cm	98 kg	37.4	52 kg	24 month
P11	23	Female	Bachelor degree	Single	Nurse	1.60 cm	123 kg	48.0	75 kg	16 month
P12	47	Male	High school	Married	Tradesman	1.73 cm	218 kg	72.8	87 kg	24 month
P13	48	Female	Secondary school	Married	Housewife	1.60 cm	122 kg	47.7	65 kg	18 month

### 2.4 Data collection tools

Two data collection tools were used in the study: a Participant Information Form and a Semi-Structured Interview Guide.

Participant Information Form: This form was developed to collect participants’ sociodemographic and clinical characteristics, including age, gender, marital status, education level, occupation, height, preoperative weight, current weight, and time elapsed since surgery.

Semi-Structured Interview Guide: The guide consisted of open-ended questions developed based on the relevant literature and reviewed by experts for content appropriateness. The questions addressed nutritional behaviors before and after metabolic bariatric surgery, difficulties experienced during the adaptation process, nursing education, environmental support, fear of weight regain, and strategies used to control eating behavior. The same Semi-Structured Interview Guide was used for both the FGDs and ISIs to ensure consistency in the core topics explored across the two data-collection methods. Follow-up probes were used when necessary to clarify or further explore participants’ responses.

### 2.5 Data collection procedure

Data were collected in two sequential stages, beginning with FGDs and followed by ISIs with the same participants. Four FGDs were conducted, with three to four participants in each group (3, 3, 3, and 4 participants, respectively). Participants were allocated to the groups according to their availability for the scheduled dates and times. The relatively small group size facilitated active participation and provided each participant with sufficient opportunity to describe and discuss their experiences in depth. Following the FGDs, ISIs were conducted to enable participants to elaborate on their individual experiences and to clarify or further explore issues that emerged during the group discussions.

All FGDs and ISIs were conducted by the first author, who had received training in qualitative research methods and interviewing techniques. No prior relationship existed between the researcher and the participants before recruitment. Prior to data collection, participants were informed about the purpose of the study, the data collection procedures, the use of audio recording, the approximate duration of the FGDs and ISIs, confidentiality principles, and their right to withdraw from the study or discontinue recording at any time. Written informed consent was obtained from all participants.

To enhance accessibility and participation, all FGDs and ISIs were conducted online via Zoom in a private setting. Each FGD lasted at least 30 min (14), with an average duration of approximately 54 min, whereas each ISI lasted approximately 20–30 min. With participants’ permission, all FGDs and ISIs were audio-recorded and subsequently transcribed verbatim by the researchers using Microsoft Word.

Member checking was performed to enhance the credibility of the findings. Participants were invited to review and confirm the accuracy of the researchers’ interpretations to ensure that these interpretations adequately reflected their experiences and perspectives.

### 2.6 Data saturation

Data saturation was assessed concurrently with data collection. During the fourth FGD, participants’ accounts increasingly repeated information obtained in the preceding groups, and no new information relevant to the developing codes and categories emerged. Therefore, saturation of the FGD data was considered to have been reached, and no additional focus groups were conducted. The subsequent ISIs did not yield new information requiring the development of additional codes or categories, further supporting the assessment that data saturation had been achieved.

### 2.7 Data analysis

The FGD and ISI transcripts were initially read and coded separately to preserve the distinct characteristics of each data source. During the analysis of the FGD data, particular attention was paid to group interactions, including instances in which participants supported, elaborated on, or expressed differing views in response to one another, thereby capturing the interactive nature of the focus group data. The source of each code was tracked throughout the analysis to determine whether findings originated from the FGD data, the ISI data, or both. The two datasets were subsequently compared and integrated during the later stages of analysis to identify areas of convergence and complementarity.

The data were analyzed using an inductive qualitative content analysis approach following the methodological principles described by Graneheim and Lundman (21). To facilitate systematic data management, coding, and organization, all transcripts were imported into MAXQDA 2022 (VERBI Software, Berlin, Germany). The transcripts were read repeatedly to achieve familiarity with the data. Meaning units relevant to the research aims were identified and condensed while preserving their core meaning, and initial codes were assigned. Codes were continuously compared and grouped according to conceptual similarities to form categories. Related categories were then organized into subthemes, and conceptually related subthemes were grouped into overarching themes.

Initial coding was conducted by the first researcher and independently reviewed by a second researcher experienced in qualitative research. Discrepancies in coding, category development, and the development of subthemes and themes were discussed until consensus was reached. An intercoder agreement of at least 80% was considered acceptable. The final themes and subthemes were interpreted in relation to the study objectives. The study was reported in accordance with the Consolidated Criteria for Reporting Qualitative Research (COREQ) checklist to enhance transparency and completeness in reporting.

### 2.8 Ethical considerations

Before the study was initiated, ethical approval was obtained from the Karabuk University Non-Interventional Research Ethics Committee (Decision No. 2021/539, Approval No. E-77192459-050.99-23987, Date: 7 April 2021). All procedures were conducted in accordance with the ethical standards of the Declaration of Helsinki. Participants were informed about the aim and scope of the study, and written informed consent was obtained from all participants before the FGDs and ISIs. Participation in the study was voluntary, and participants were informed that they could withdraw from the study at any stage without any consequences. Confidentiality and anonymity of the participants were ensured throughout the research process, and the collected data were used solely for scientific purposes.

## 3 Results

### 3.1 Participant characteristics

Participants were aged 23–48 years. Of the participants, 11 were female and two were male, and 11 were married. Educational levels ranged from secondary school to a master’s degree, and participants represented diverse occupational backgrounds, including nursing, academia, law, engineering, and other occupations. Participants’ preoperative body weight ranged from 98 to 218 kg, whereas their current body weight ranged from 52 to 87 kg. Their height ranged from 1.50 to 1.79 m, and the time elapsed since metabolic bariatric surgery ranged from 15 to 24 months. Detailed demographic and clinical characteristics of the participants are presented in Table 1.

Qualitative content analysis resulted in five overarching themes reflecting participants’ experiences before and after metabolic bariatric surgery: (1) Decision-Making Process for Metabolic Bariatric Surgery, (2) Pre-Surgical Nutritional Patterns, (3) Strategies for Nutritional Adaptation After Metabolic Bariatric Surgery, (4) Postoperative Challenges Following Metabolic Bariatric Surgery, and (5) Information Sources and Educational Support During the Surgical Process. Together, these themes illustrate participants’ experiences from the preoperative decision-making and nutritional period through postoperative nutritional adaptation, associated challenges, and the educational and informational support received throughout the surgical process. The findings for each theme and its corresponding subthemes are presented below.

### 3.2 Theme 1. Decision-making process for metabolic bariatric surgery

**FIGURE 1:**
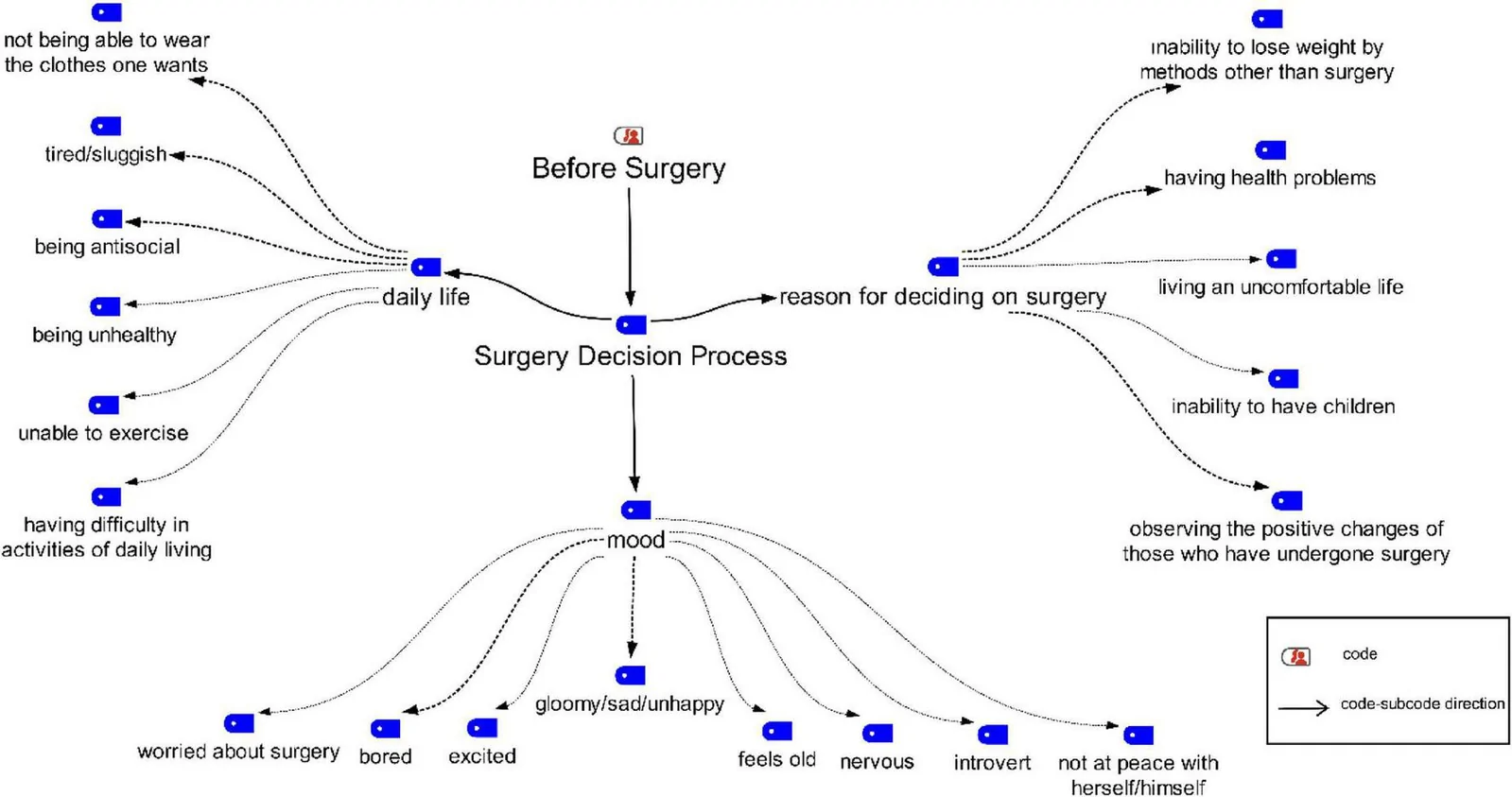
Conceptual representation of factors influencing participants’ decision-making for metabolic bariatric surgery.

Participants described the decision to undergo metabolic bariatric surgery as a multidimensional process shaped by limitations in daily life, psychological difficulties, and multiple factors influencing the choice of surgery. Figure 1 presents the conceptual relationships among the codes generated through qualitative content analysis and illustrates the factors shaping participants’ decisions to undergo metabolic bariatric surgery.

#### 3.2.1 Daily life limitations

Participants reported that obesity negatively affected multiple aspects of their daily lives. They described persistent fatigue, difficulties performing activities of daily living, limitations in clothing choices and participation in physical activities, reduced social engagement, and perceptions of poor physical health.

“*Starting the day tired and feeling increasingly weary throughout the day was taking away all my joy. I was getting very tired and struggling even to meet my personal needs, which saddened me greatly and also made me think about my later years. I thought I wasn’t a good servant of God, a good wife, or a good mother. My social activities decreased day by day, even though I used to be a hardworking and sociable person; now, even if I wanted to, I couldn’t because I had no energy.” (P7/48/Female/Married/Bachelor Degree/Engineer)*

“*Before metabolic bariatric surgery, I always felt like I was missing something. For example, I couldn’t dress like my peers; I always had to choose clothes that fit me, not what I wanted or liked. That’s why I didn’t like standing up much in social situations.” (P9/44/Female/Married/High School/Housewife)*

#### 3.2.2 Psychological impact

Participants described various psychological difficulties before surgery, including sadness, unhappiness, tension, boredom, social withdrawal, dissatisfaction with themselves, feeling older than their age, and anxiety related to the surgical procedure.

“*Before the surgery, I felt much more tired, sleep-deprived, unhappy, weak, tense about everything, negative, and incapable of solving any problems in my life.” (P10/45/Female/Married/High School/Coordinator)*

#### 3.2.3 Reasons for choosing surgery

Participants identified several factors that influenced their decision to undergo metabolic bariatric surgery, including obesity-related health problems, unsuccessful previous attempts to lose weight through non-surgical methods, reduced quality of life, concerns related to infertility, and positive outcomes observed among individuals who had previously undergone the procedure.

“*My kidneys were constantly producing stones, and I needed to have them broken up. Unfortunately, I couldn’t get on the operating tables alone. I needed an MRI, but I couldn’t go in. I wasn’t worried about my clothes, but I started having serious health problems. I had high blood pressure, and when my blood pressure reached 240/260, my husband and I talked, and I realized I had no other option. I made the decision because I had many health problems.” (P13/48/Female/Married/Secondary School/Housewife)*

Overall, participants’ accounts indicate that the decision to undergo metabolic bariatric surgery emerged from the interplay of limitations in daily life, psychological difficulties, obesity-related health concerns, previous weight-loss experiences, and expectations of improved health and quality of life.

### 3.3 Theme 2. Pre-surgical nutritional patterns

**FIGURE 2:**
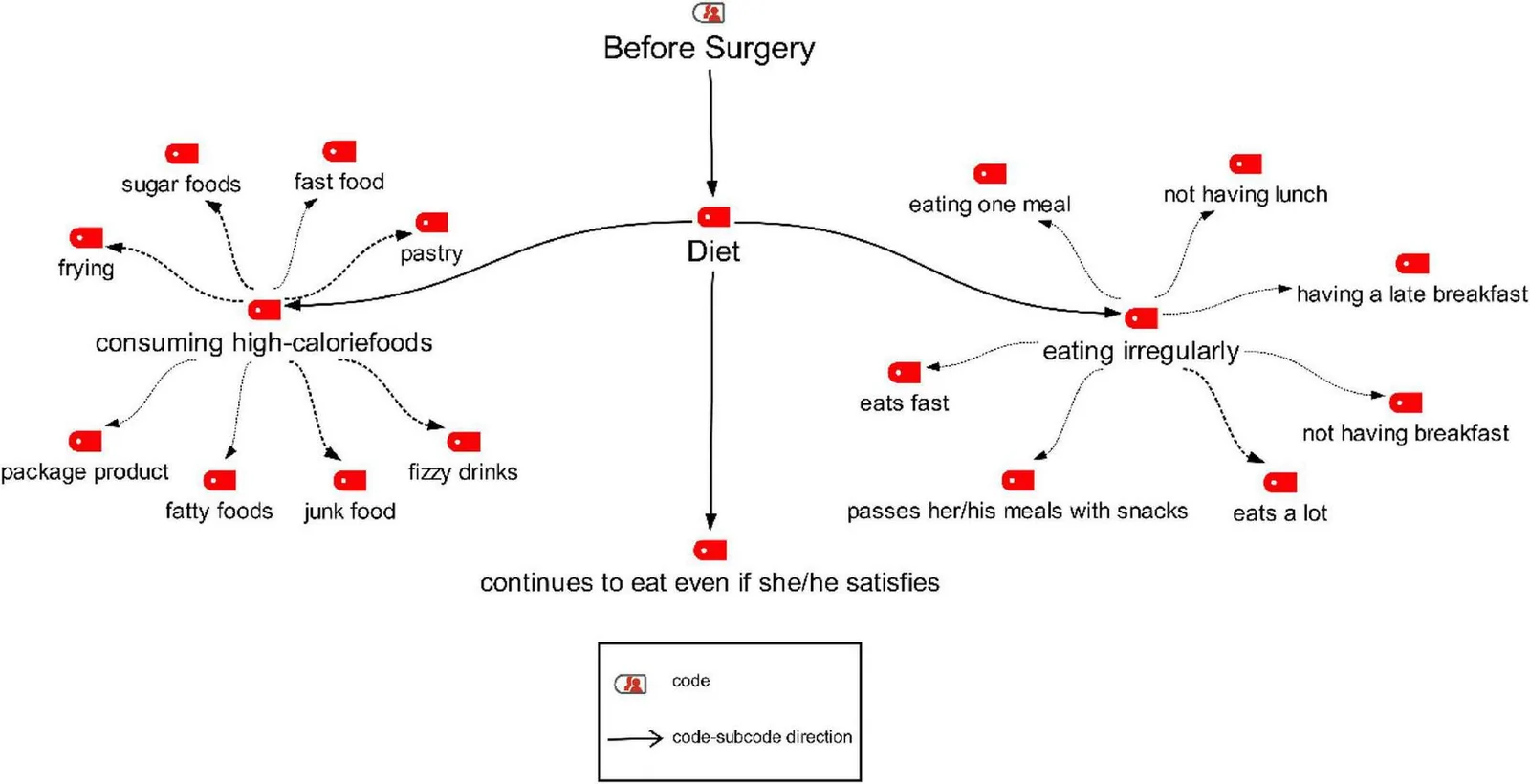
Conceptual representation of participants’ pre-surgical nutritional patterns.

Participants described their pre-surgical nutritional patterns as being characterized by frequent consumption of high-calorie foods and irregular eating behaviors, including difficulties maintaining regular meal patterns and regulating food intake. Figure 2 presents the conceptual representation of the pre-surgical nutritional patterns identified through qualitative content analysis.

#### 3.3.1 Consuming high-calorie foods

Participants frequently reported consuming high-calorie foods and beverages, including sugary products, pastries, fried and fatty foods, fast food, carbonated beverages, and packaged snacks. These dietary practices were described as long-standing habits that participants associated with their preoperative weight gain.

“*Every day I would drink cola, eat pastries and savory pies, and consume fried foods.” (P11/23/Female/Single/Bachelor Degree/Nurse)*

“*Perhaps my biggest mistake was being addicted to junk food, like chocolate. Chocolate was indispensable to me; I even had chocolate cravings. I would only leave the house to go to the market to buy chocolate.” (P3/30/Female/Married/Master’s Degree/Academic)*

“*I was eating whatever I wanted, consuming a lot of cola and junk food.” (P6/30/Female/Married/Bachelor Degree/Lawyer)*

#### 3.3.2 Eating irregularly

In addition to unhealthy food choices, participants described irregular eating patterns characterized by skipping meals, consuming only one main meal per day, replacing meals with snacks, eating rapidly, consuming large portions, and continuing to eat despite feeling full.

“*For breakfast, I would eat carbohydrate-heavy foods like bagels, pastries, rolls, and bread, along with large amounts of cheese and processed meats… I used to gain weight constantly because I ate quickly, consumed a lot of carbohydrates and fatty foods, and high-calorie foods.” (P5/41/Male/Married/Bachelor Degree/Tradesman)*

“*For breakfast, there is always one of these four: toast, bagel, pastry, or bread… Weekend breakfasts include additions such as french fries, menemen, börek, kuymak, etc.” (P7/48/Female/Married/Bachelor Degree/Engineer)*

Overall, participants’ accounts indicate that pre-surgical nutritional patterns involved the combined influence of unhealthy food choices, irregular meal routines, and difficulties regulating food intake.

### 3.4 Theme 3. Strategies for nutritional adaptation after metabolic bariatric surgery

**FIGURE 3:**
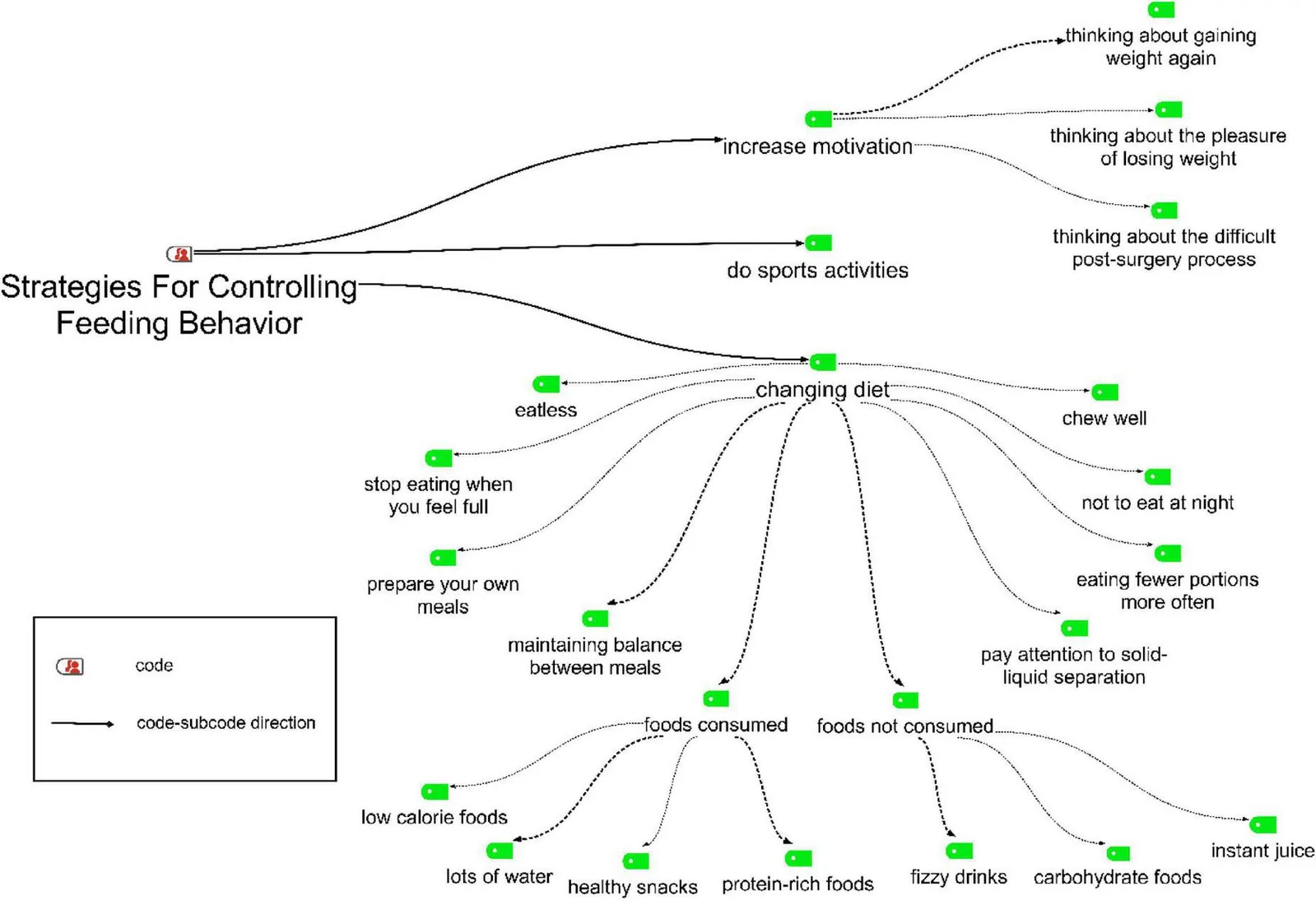
Conceptual representation of participants’ strategies for nutritional adaptation after metabolic bariatric surgery.

Participants described nutritional adaptation following metabolic bariatric surgery as being supported by both external and individual strategies, particularly social support and behavioral and dietary self-regulation. Figure 3 presents the conceptual representation of the strategies identified through qualitative content analysis.

#### 3.4.1 Social support

Participants emphasized the importance of family members and close friends in facilitating adherence to healthy eating behaviors after surgery. Family members adapted household eating practices, avoided consuming unhealthy foods in participants’ presence, and provided emotional encouragement throughout the adaptation process. Friends also contributed by encouraging adherence to dietary recommendations and accommodating participants’ nutritional needs during social gatherings.

“*My wife was very helpful too, she gave it up. My son was a picky eater, but I didn’t prepare separate meals for him. We ate mostly vegetable-based meals, soups and things like that. They helped me at home, didn’t let me cook out, and supported me. They consumed packaged foods when I wasn’t home or when I was out so I wouldn’t crave them. They were very helpful in this regard.” (P13/48/Female/Married/Secondary School/Housewife)*

“*I received a lot of help, especially from my family and close friends; they gave me encouragement to get through this process easily, tried to keep me focused on my goal, and supported me to increase my patience. It was a great help that the treats offered when we met with my friends were the kind I could eat.” (P7/48/Female/Married/Bachelor Degree/Engineer)*

#### 3.4.2 Behavioral and dietary self-regulation

Participants described several self-regulatory strategies for managing their nutritional behaviors after surgery. These included avoiding high-calorie foods, monitoring food intake, increasing water consumption, controlling portion sizes, and adjusting subsequent meals when they perceived that they had overeaten.

“*I try to eat carefully and avoid high-calorie foods. I make sure to drink plenty of water. I don’t overeat because I’m afraid of gaining weight again; I pay attention to calories, and if I realize I’ve eaten too much, I make my next meal lighter.” (P6/30/Female/Married/Bachelor’s Degree/Lawyer)*

“*If my water level is low, I increase it. I protect myself with these kinds of changes.” (P8/35/Female/Married/High School/Housewife)*

“*I try to avoid high-calorie foods and I try to control myself in this regard.” (P10/45/Female/Married/High School/Coordinator)*

Overall, participants’ accounts indicate that postoperative nutritional adaptation involved a combination of social support and individual efforts to monitor and regulate dietary behaviors.

### 3.5 Theme 4. Postoperative challenges following metabolic bariatric surgery

**FIGURE 4:**
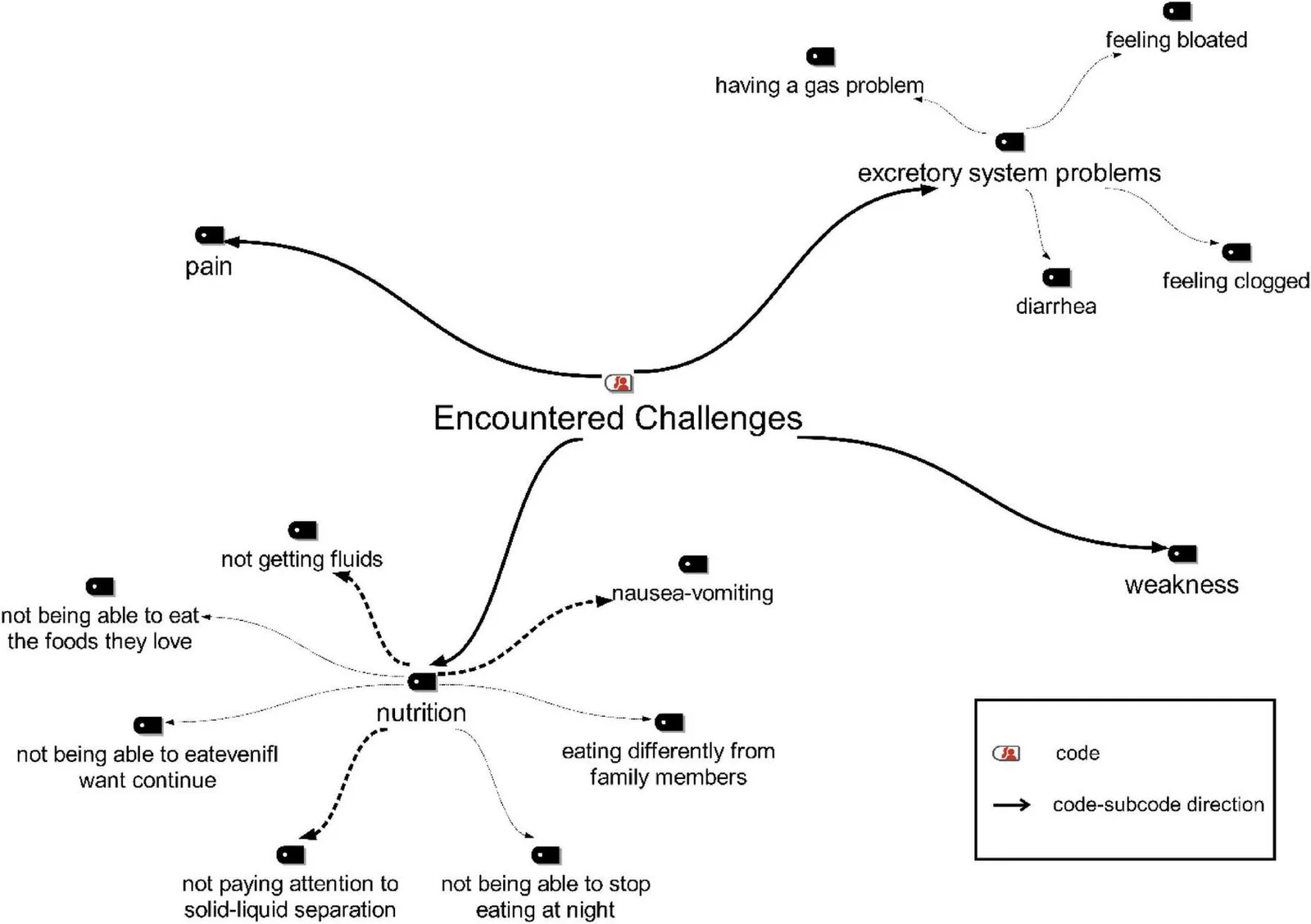
Conceptual representation of postoperative challenges experienced by participants.

Participants described postoperative adaptation as a complex process involving physical, behavioral, psychological, and social challenges that required continuous adjustment to new dietary practices and lifestyle changes. Figure 4 presents the conceptual representation of the postoperative challenges identified through qualitative content analysis.

#### 3.5.1 Physical challenges

Participants described the early postoperative period as physically challenging, particularly during the first weeks following surgery. Commonly reported difficulties included nausea, vomiting, early satiety, stomach sensitivity, and fatigue.

“*I would describe the first three months as the most challenging part. Especially the first week and the first month are probably the most brutal period after this surgery. Starting with fluid intake on the morning of surgery, you deeply experience the psychological difficulty of taking the first step into a period so challenging that you can’t even consume fluids. I could only drink a glass of water after 40–45 minutes.” (P1/32/Female/Single/High School/Nurse)*

“*The first three months were very difficult for me due to nausea and vomiting. Because I didn’t know my stomach capacity exactly, I was throwing up the excess.” (P6/30/Female/Married/Bachelor Degree/Lawyer)*

#### 3.5.2 Challenges in eating behavior

Participants described difficulties adapting to new eating behaviors after surgery, including modifying previous eating habits, chewing food adequately, slowing the pace of eating, controlling portion sizes, and avoiding overeating.

“*Yes, I experienced some problems. Of course, since I wasn’t used to it, sometimes I would vomit if I ate without chewing properly, sticking to my old habit. I was very afraid of exceeding the recommended amount.” (P7/48/Female/Married/Bachelor Degree/Engineer)*

“*I’ve had a lot of muscle spasms due to eating too fast and not chewing properly.” (P2/33/Female/Married/High School/Nurse)*

“*I’m trying to stop eating at night; if I stop for a week, I start eating again on the third day. I haven’t been able to cut down on night eating completely.” (P13/48/Female/Married/Secondary School/Housewife)*

#### 3.5.3 Psychological challenges

Participants described psychological challenges during postoperative adaptation, including anxiety related to recovery, fear of exceeding recommended food intake, concerns about weight regain, and the emotional demands of adjusting to a new lifestyle.

“*…I was very afraid of exceeding the recommended amount.*” (P7/48/Female/Married/Bachelor Degree/Engineer)

#### 3.5.4 Social and environmental challenges

Participants described difficulties maintaining their new nutritional routines within the family and home environment, particularly when other family members continued their usual eating practices.

“*Honestly, the process at home is actually more difficult. Because everyone except me had to continue with their normal meal routines.*” (P1/32/Female/Single/High School/Nurse)

Overall, participants’ accounts indicate that postoperative adaptation involved interconnected physical, behavioral, psychological, and social challenges that required ongoing adjustment to dietary and lifestyle changes.

### 3.6 Theme 5. Information sources and educational support during the surgical process

**FIGURE 5:**
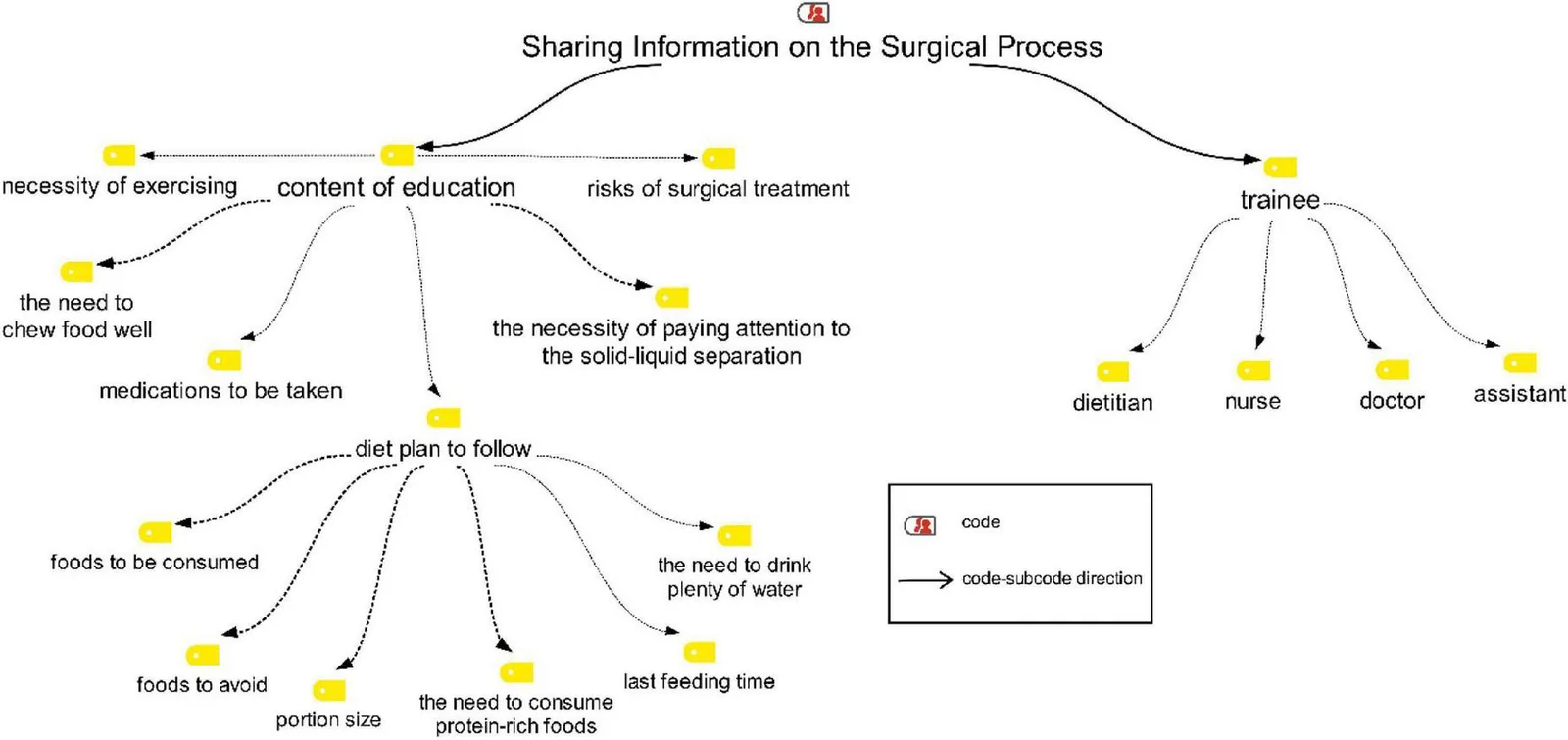
Conceptual representation of information sources and educational support during the surgical process.

Participants described receiving information and educational support throughout the surgical process from both healthcare professionals and informal sources. These sources provided guidance related to postoperative care, nutritional management, and adaptation to lifestyle changes. Figure 5 presents the conceptual representation of the information sources and educational support identified through qualitative content analysis.

#### 3.6.1 Professional education and counseling

Participants reported receiving education and counseling from nurses, physicians, and dietitians before and after surgery. The educational content primarily addressed dietary progression, fluid intake, meal planning, medication use, exercise, and the gradual transition from liquid to solid foods. Participants also described receiving written educational materials and follow-up counseling to reinforce postoperative recommendations.

“*First, they provided training on what to eat and drink in the hospital, what to pay attention to during meals, and how much to consume, and then they also gave me a written document. I went for a check-up on the 15th day after the surgery, and they adjusted my meal plan then. Similarly, in the first, second, and third months, I received training and support on which foods to add to my diet each month.*” (P3/30/Female/Married/Master’s Degree/Academic)

“*Since it wasn’t a very well-known surgery, they just mentioned general things. I don’t think there were many details; things like separating solids from liquids, carbonated drinks, etc., meaning we shouldn’t eat too many carbohydrates and we should exercise. The doctor, nurse, and dietitian all said these things.*” (P8/35/Female/Married/High School/Housewife)

“*After the surgery, I was told that I would first be fed liquids, then purees, and then gradually solids. The importance of chewing thoroughly, the distinction between liquids and solids, the need for exercise, and the importance of a low-calorie but high-protein diet were explained.*” (P5/41/Male/Married/Bachelor Degree/Tradesman)

#### 3.6.2 Informal sources of information and experience sharing

In addition to information provided by healthcare professionals, participants described obtaining information from informal sources, particularly family members and individuals who had previously undergone metabolic bariatric surgery. These sources provided experiential knowledge about the surgical process and reassurance during decision-making.

“*This surgery was mainly recommended to me because my wife had previously undergone the procedure with the same physician. At first, I was hesitant, but after meeting him personally, I eventually became convinced to undergo the surgery.*” (P12/47/Male/Married/High School/Tradesman)

Overall, participants’ accounts indicate that information and educational support were obtained from both healthcare professionals and informal sources, providing complementary forms of guidance throughout the surgical process.

## 4 Discussion

Participants in the present study described the decision to undergo metabolic bariatric surgery as being shaped by the cumulative physical, psychological, and social consequences of obesity. Social withdrawal, persistent fatigue, reduced physical functioning, negative self-perception, and unsuccessful weight-loss attempts collectively contributed to their consideration of surgical treatment. This finding is consistent with qualitative evidence showing that individuals living with obesity may perceive bariatric surgery as their “only hope” for restoring health and improving daily functioning (22). Similarly, previous qualitative syntheses have demonstrated that bariatric surgery is often viewed as an opportunity to regain control over life and re-establish a sense of normality after years of living with obesity (3). Psychological distress has also been frequently reported among bariatric surgery candidates (23). In the present study, however, participants described not only anxiety and uncertainty but also hope and excitement, reflecting a complex emotional transition during the decision-making process. Weight-related stigma and the internalization of negative societal attitudes toward obesity may further intensify emotional distress and shape individuals’ experiences when considering surgical treatment (24, 25). These observations underscore the importance of comprehensive preoperative assessment that considers both the physical and psychosocial consequences of obesity.

Unsuccessful weight-loss attempts and obesity-related health problems emerged as important factors in participants’ decisions to undergo metabolic bariatric surgery. This observation is consistent with evidence indicating that individuals often consider surgery after conservative weight-management approaches have been unsuccessful and obesity has substantially affected their health and quality of life (2). Several female participants also identified reproductive health concerns as a factor in their decision to undergo surgery. Previous studies have similarly reported that women of reproductive age may consider bariatric surgery to improve fertility, reduce pregnancy-related risks, and optimize maternal health before conception (26). These observations highlight the importance of individualized preoperative education and counseling that considers patients’ personal expectations, long-term goals, and readiness for behavioral change. Within the multidisciplinary team, nurses can contribute by assessing patients’ educational and psychosocial needs, supporting informed decision-making, and coordinating preoperative care.

Participants described their preoperative eating patterns as being characterized by frequent consumption of energy-dense foods, irregular meal patterns, and difficulties regulating food intake. Similar dietary patterns have been reported among individuals seeking metabolic bariatric surgery (27). Emotional and habitual eating may further complicate the regulation of food intake beyond physiological hunger and create challenges for long-term dietary modification (28). These observations underscore the importance of assessing maladaptive eating behaviors before surgery and providing individualized nutritional counseling to support sustainable behavioral change.

Participants described postoperative nutritional adaptation as an ongoing process requiring dietary and behavioral adjustments. Strategies such as portion control, eating slowly, adapting to reduced gastric capacity, meal planning, and self-monitoring were described as important components of establishing and maintaining new nutritional routines. This observation is consistent with qualitative evidence indicating that patients gradually reconstruct their daily routines around self-regulation, control, and the restoration of normality following bariatric surgery (3). Sustained behavioral interventions and self-monitoring have also been associated with more favorable long-term weight outcomes and a reduced risk of weight regain (29, 30). These observations suggest that long-term nutritional adaptation involves not only the physiological effects of surgery but also the development and maintenance of behavioral self-regulation strategies.

Participants described postoperative adaptation as a multidimensional process involving physical symptoms, dietary adjustments, psychological concerns, and social circumstances. Gastrointestinal symptoms, reduced food tolerance, and difficulties adapting to new eating behaviors were particularly evident during the early postoperative period, consistent with previous studies examining nutritional adequacy and food intolerance following bariatric surgery (31–33). Participants also expressed concerns about weight regain and described social support as an important aspect of the adaptation process. Previous research has similarly identified behavioral, psychological, physiological, and environmental factors in relation to weight regain and has highlighted the relevance of social support to long-term postoperative adaptation (34, 35). These observations underscore the importance of structured long-term follow-up after metabolic bariatric surgery. Within the multidisciplinary team, nurses can contribute to this process by monitoring postoperative adaptation, reinforcing nutritional recommendations, identifying emerging behavioral difficulties, and facilitating access to appropriate support.

Participants emphasized the importance of receiving clear and consistent information throughout the surgical process for understanding postoperative care and adapting to dietary and lifestyle changes. Previous studies have shown that educational interventions can improve patients’ knowledge, self-care behaviors, and adherence to postoperative recommendations (36). Bariatric surgery candidates may also require individualized and ongoing information throughout the treatment process, as unmet informational needs have been associated with uncertainty and anxiety (37). Although participants also obtained information from family members and individuals who had previously undergone bariatric surgery, such experiential knowledge may be most valuable when used to complement rather than replace professional guidance (3). These observations underscore the importance of individualized and continuous patient education throughout the perioperative period.

Taken together, the findings suggest that adaptation following metabolic bariatric surgery extends beyond the surgical procedure and encompasses interconnected nutritional, behavioral, psychological, and social dimensions across the preoperative and postoperative periods. Participants’ experiences underscore the importance of patient-centered and individualized care that integrates nutritional management with emotional preparation, behavioral support, and long-term follow-up. Within a multidisciplinary approach, nurses can contribute to this process through patient education, ongoing monitoring, early identification of support needs, care coordination, and reinforcement of sustainable lifestyle behaviors throughout the metabolic bariatric surgery journey.

## 5 Strengths and limitations

Several limitations should be considered when interpreting the findings of this study. First, the relatively small sample size may limit the transferability of the findings, although this is consistent with the in-depth and exploratory nature of qualitative research. Second, the study involved participants who had undergone metabolic bariatric surgery at a single hospital, which may limit the transferability of the findings to individuals receiving care in different healthcare settings. In addition, participants were recruited using purposive sampling and shared a similar regional and cultural context, which may have limited socioeconomic and experiential diversity. Although no educational criterion beyond literacy was intentionally used to restrict participation, all participants had at least a secondary level of education. Therefore, the experiences of individuals with lower educational attainment may not be adequately represented. Furthermore, the findings were based on participants’ retrospective accounts and may have been influenced by recall bias. Some participants were healthcare professionals, whose professional backgrounds may also have influenced how they interpreted and articulated their experiences as patients.

Despite these limitations, the study has several strengths. The use of both FGDs and ISIs provided complementary perspectives on participants’ experiences, allowing shared experiences emerging through group interaction to be considered alongside more detailed individual accounts. The study also provides a comprehensive account of the metabolic bariatric surgery experience across multiple stages, from preoperative decision-making and nutritional behaviors to postoperative challenges, adaptation strategies, and educational support. By integrating these dimensions, the findings contribute to a more nuanced understanding of how individuals experience and manage nutritional and lifestyle changes following metabolic bariatric surgery. The findings may also contribute to nursing knowledge by identifying areas in which individualized education, social support, and continued follow-up may be particularly relevant throughout the perioperative and postoperative adaptation process.

## 6 Conclusion

The findings of this study indicate that adaptation following metabolic bariatric surgery extends beyond the surgical procedure and encompasses nutritional, behavioral, psychological, and social dimensions. Participants’ experiences highlight the need for sustained support in adapting to dietary changes, managing postoperative challenges, and establishing new lifestyle behaviors. Multidisciplinary care, including nursing education, counseling, monitoring, and follow-up, is important in addressing patients’ evolving needs throughout the surgical process. Individualized preoperative education and long-term follow-up may support postoperative adaptation and the maintenance of sustainable nutritional and lifestyle behaviors.

## Data Availability

The raw data supporting the conclusions of this article will be made available by the authors, without undue reservation.
